# Stress–Strain Hysteresis Loop-Based Machine Learning Models for Predicting Metal Fatigue Life Under Uncertainty

**DOI:** 10.3390/ma18184336

**Published:** 2025-09-16

**Authors:** Xian-Ci Zhong, Zhi-Yong Luo, Ke-Shi Zhang

**Affiliations:** 1 School of Civil Engineering and Architecture, Guangxi University, Nanning 530004, China; 2210303010@st.gxu.edu.cn (Z.-Y.L.); zhangks@gxu.edu.cn (K.-S.Z.); 2 Key Laboratory of Disaster Prevention and Structural Safety of Ministry of Education, Guangxi University, Nanning 530004, China

**Keywords:** fatigue life prediction, stress–strain hysteresis loop, polar coordinate, machine learning, uncertainty

## Abstract

This paper reports machine learning models for predicting metal fatigue life under uncertainty by extracting stress–strain data from hysteresis loops. First, the hysteresis loops of Q235B under strain-controlled constant amplitude loading are analyzed. The values of stress and strain in six key points are extracted from each hysteresis loop at the earliest stages of the fatigue process, and transformed into polar coordinates. Second, the uncertainty is quantified by extending the applied strain amplitude and the selected stress–strain values to intervals. A great deal of data are generated randomly in each interval for coping with the challenge of a small fatigue test dataset. Third, three machine learning models are constructed, where the parameters of the back-propagation neural network model are optimized by using the leave-one-out cross-validation technique, and the models of support vector regression and random forest are selected carefully. The point and interval predictions of the low-cycle-fatigue life of Q235B are reported to reveal the feasibility and advantage of the proposed models. The results help to identify how to understand the fatigue behavior of materials by combining machine learning models and stress–strain hysteresis loops.

## 1. Introduction

In practical engineering, fatigue is one of the key factors leading to component damage and failure [1]. To reasonably evaluate the service life of components, it is crucial to have a deep understanding of the fatigue mechanism and to construct a reliable fatigue life-prediction model [2]. Since metal is a commonly used engineering material, it is interesting to explain fatigue behaviors and predict fatigue life of metallic materials [3,4]. Recently, machine learning (ML) models have been used widely to predict metal fatigue life [5,6,7]. It is worth noting that the stress–strain hysteresis loop is an important foundation for characterizing and studying the fatigue behavior of metallic materials under cyclic loading. Thus, in the present study we develop ML models driven by reasonably selecting stress–strain data of hysteresis loops. This constitutes a novel attempt to understand the fatigue behavior of metallic materials according to a combination of ML techniques and hysteresis loops.

In machine learning models for predicting metal fatigue life, one of the important issues is to construct the training set. Initially, the training set is made by only considering the geometric and loading factors of fatigue specimens. For example, the geometric and loading data of 10 specimens with various welding defects were used to train a neural network for evaluating the fatigue life in [8]. The parameters of shot peening process and materials together with stress amplitude were considered to train a back-propagation neural network to predict the fatigue life of carbon steels in [9]. The location and size of critical defects inside additively manufactured metals were measured by using the high cycle fatigue postmortem examination and synchrotron X-ray tomography, and combined with the morphology of specimens to train a support vector machine (SVM) for fatigue life prediction in [10]. Additive manufacturing process parameters and fatigue loadings were used as the input data of machine learning models for predicting the fatigue life of printed SS 316L with defects in [11]. Fatigue, creep and creep-fatigue data of 316 stainless steel were integrated to train three machine learning models and a deep neural network for life prediction in [12]. For predicting multiaxial fatigue life, the training set was composed of the data derived from the multiaxial path and temperature in [13].

It is seen that the above-mentioned works only focus on the point predictions of fatigue life based on a fatigue test dataset. There are two other questions that should be addressed. One is that fatigue test datasets are usually small due to cost and time constraints. This means that ML models may not have been adequately trained. The other is the dispersion of fatigue life under uncertainty [14], which cannot be characterized according to point predictions. The former is the challenge of small fatigue test datasets, which can be coped with to a certain degree by augmenting and/or expanding the fatigue test dataset. For instance, the initial fatigue dataset was augmented by using the methods of inverse transform sampling and multivariate radial basis function (RBF) interpolation in [15]. A damage mechanics model was adopted to generate data such that the extreme gradient boosting (XGBoost) ML model was trained to predict the high-cycle-fatigue life of ZM6 in [16]. A great deal of data were randomly generated to expand the fatigue test dataset for training ML models in [17,18]. Moreover, it is revealed that physics-informed neural network models exhibit the advantages of addressing the challenge of small dataset [19,20,21]. The latter is the challenge of dispersion to fatigue life prediction, which can be addressed by considering uncertainty quantification to some extent [17,18,22]. Thus, the statistical distribution, mean, and standard variance of fatigue life have been predicted [23,24,25,26,27,28].

In addition, the fatigue experiments of metallic materials show that the stress–strain hysteresis loop is an important characterization of the fatigue process. The analysis of hysteresis loops contributes to the explanation of the fatigue mechanism and the prediction of fatigue life [29,30]. The data of force peaks at the earliest stages of the fatigue process have been extracted to train ML models and improve the accuracy of fatigue life predictions [17,18]. Lots of hysteresis-loop data were adopted in an ML model for predicting the low-cycle-fatigue life of wrought Mg alloys [31], where five features of 548 hysteresis loops were considered such as the loading direction, strain amplitude, number of loading cycles, strain in a hysteresis loop, and corresponding stress. To address the challenges of small a fatigue test dataset and uncertainty in fatigue life prediction, there are some research gaps in combining the data of hysteresis loops and ML techniques.

(1) Hysteresis loop reflects the energy dissipation and plastic-deformation behavior of materials under alternating stress. There is a close relationship between hysteresis loops and fatigue life. How can data be selected from each hysteresis loop such that the features affecting fatigue life predictions can be extracted? Although the stress–strain data of hysteresis loops have been considered in predicting fatigue life [31], the above-mentioned question should still be investigated in depth.
(2) There are too many hysteresis loops and stress–strain values in a hysteresis loop. How many hysteresis loops and stress–strain values should be selected to train ML models for reasonably predicting metal fatigue life? It is seen from the existing works in [17,18] that the earlier 200 cycles have been considered with a step size. When considering multiple stress–strain data, the number of hysteresis loops should still be addressed carefully.
(3) Uncertainty inevitably exists in geometry, environment, and microstructure of specimens. How can the uncertainty be quantified by selecting the stress and strain data from hysteresis loops such that the dispersion of fatigue life can be characterized? The uncertainty has been quantified as an interval with probability distributions and the statistical property of fatigue life prediction has been considered [23,24,25,26,27]. However, when the stress–strain data are extracted from hysteresis loops, the uncertainty should still be considered for characterizing the dispersion of fatigue life.

This paper focuses on the above important issues to propose ML models for point and interval predictions of fatigue life. Some novel contributions to the literature are worth mentioning below:

(a) The influence of maximum stress, minimum stress, mean stress, and residual stress on uniaxial fatigue life is considered. The corresponding stress–strain data are selected from each hysteresis loop as the input data of ML models.
(b) It is considered that the hysteresis loops at the earliest stages of the fatigue process play an important role in predicting fatigue life. The early 10 hysteresis loops are selected and analyzed to obtain the optimal dataset.
(c) By considering multiple specimens, the selected stress–strain values from hysteresis loops are extended to intervals. A great deal of randomly generated data are used to train ML models and predict fatigue life with dispersion. In addition, the leave-one-out cross-validation (LOOCV) method is used to optimize the parameters of ML models.

The structure of this paper is as follows. Section 2 focuses on the method of constructing the dataset according to stress–strain hysteresis loops under uncertainty. In Section 3, we elaborate on the models of back-propagation (BP) neural network, support vector regression (SVR), and random forest (RF). The parameter optimizations of ML models are addressed. Section 4 reports the point prediction and interval prediction of the low-fatigue life of Q235B. Some comparisons are offered to reveal the feasibility of the developed models. The conclusion and some research directions are provided in Section 5.

## 2. Dataset Establishment

In the following, we elaborate on the method of extracting the stress–strain data from hysteresis loops according to fatigue experiments of Q235B under strain-controlled constant amplitude.

### 2.1. Experimental Data

The metallic material of Q235B steel is used to conduct fatigue tests under constant strain amplitudes. The mechanical properties and main chemical composition are given in Table 1 and Table 2 [32], respectively.

**Figure 1:**
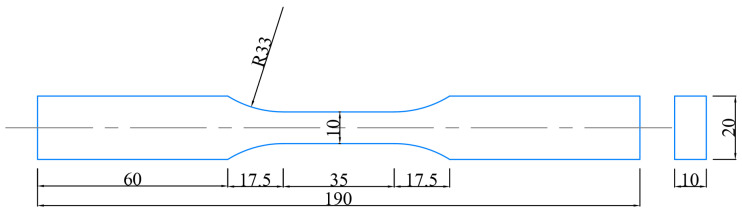
Geometry of smooth Q235B specimen (unit: mm).

The geometry of the Q235B steel smooth specimen is shown in Figure 1 [32]. Based on the standard of ASTM E466 [33], the fatigue test is carried out on the MTS809 electro-hydraulic servo tensile and torsion testing machine (MTS Systems Corporation, Eden Prairie, MN, USA) at room temperature. Under the strain-controlled constant amplitudes of 0.004, 0.005, 0.006, and 0.008, the logarithmic fatigue lives are shown in Table 3 [32], where 3 specimens are used for each strain amplitude by following the standard of ASTM E466 [33]. It should be pointed out that specimens are acted on by tensile and compressive loadings, where the loading waveform is a sinusoidal wave with a maximum frequency of 2 Hz. There is no rotation during the process of a fatigue test.

**Figure 2:**
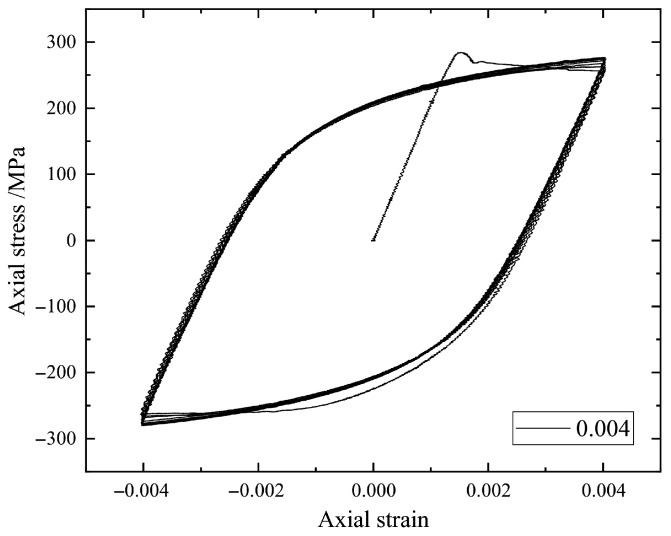
The stress–strain hysteresis loops of a specimen under strain amplitude 0.004.

**Figure 3:**
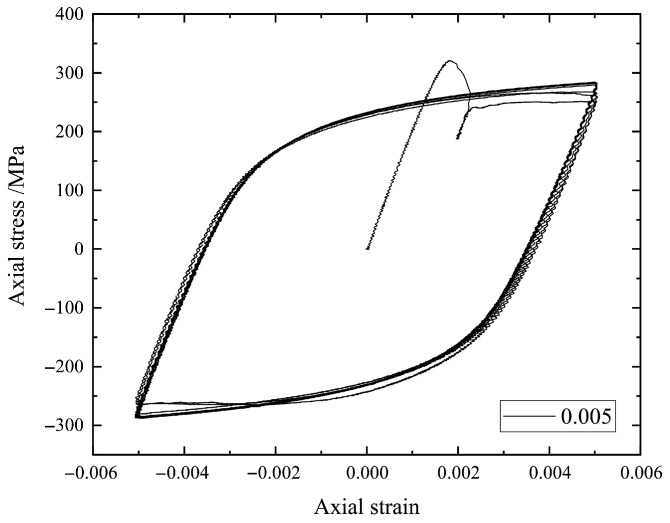
The stress–strain hysteresis loops of a specimen under strain amplitude 0.005.

**Figure 4:**
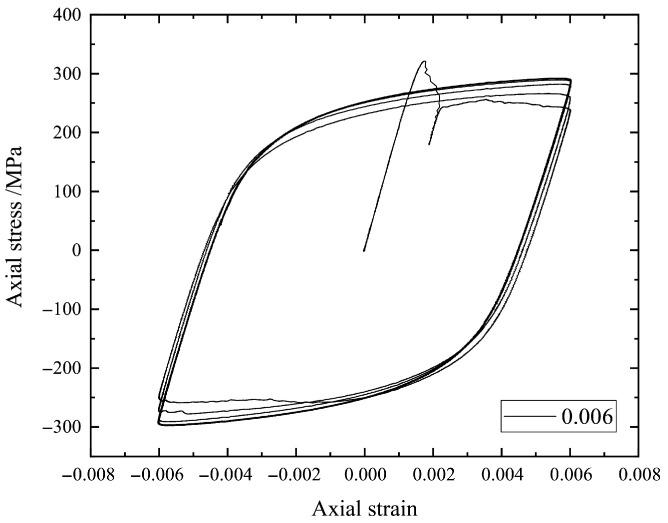
The stress–strain hysteresis loops of a specimen under strain amplitude 0.006.

**Figure 5:**
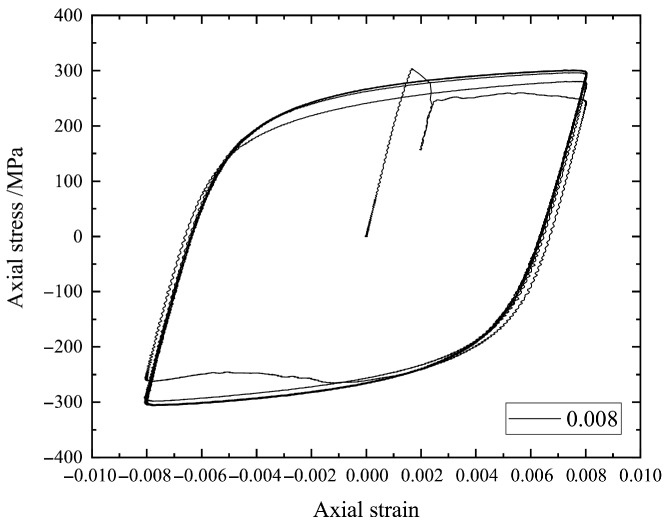
The stress–strain hysteresis loops of a specimen under strain amplitude 0.008.

Here, we focus in particular on the stress–strain hysteresis loops such as those in Figure 2, Figure 3, Figure 4 and Figure 5 by using the earliest 10 cycles. It can be seen from Figure 2, Figure 3, Figure 4 and Figure 5 that the strain amplitude is unchanged during the fatigue process under each strain loading. The stress and corresponding strain are the main physical quantities to characterize the fatigue process. The maximum stress and the minimum stress together with the mean stress are the basic parameters to describe a hysteresis loop. In the present study, the stress–strain values for describing a hysteresis loop should be carefully selected to effectively train an ML model.

### 2.2. Compilation of the Dataset

**Figure 6:**
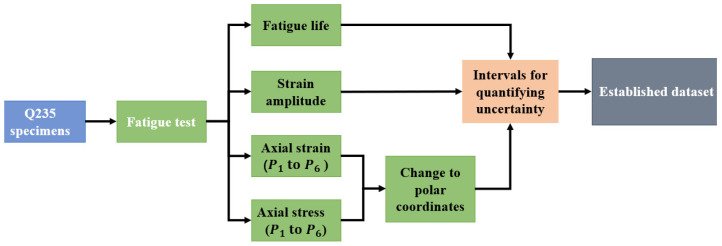
Scheme of dataset compilation process.

The establishment of a dataset depends on stress–strain hysteresis loops and uncertainty quantification. A scheme of a dataset compilation process is shown in Figure 6.

**Figure 7:**
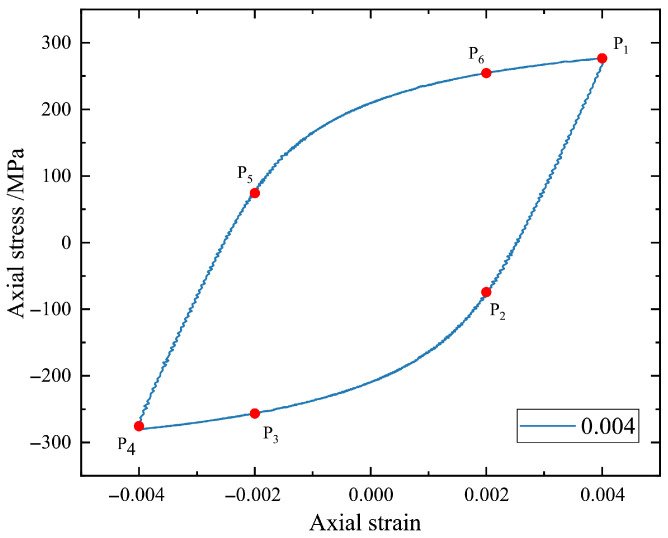
Six key points by considering the characteristics of a hysteresis loop.

**Figure 8:**
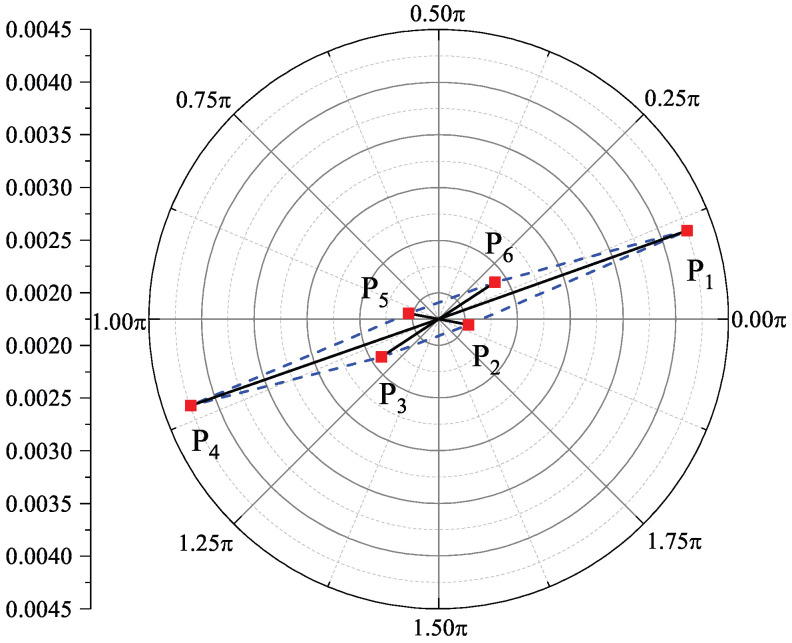
Six key points of a hysteresis loop in polar coordinates.

First, we focus on the question of how to extract data from stress–strain hysteresis loops. It is seen that the controlled strain amplitude of fatigue tests corresponds to the points of the maximum and minimum strains. This means that the two points with maximum strains should be selected. In addition, we can see from stress–strain hysteresis loops that the sudden changes in the slopes of the curves are near the points with half strain amplitude. For the sake of simplicity, the points corresponding to half strain amplitude should be selected. That is, we select six key points by considering the characteristics of hysteresis loops as shown in Figure 7. Hereafter, $P_{1}-P_{6}$ correspond to the maximum strain, half strain amplitude, negative half strain amplitude, and minimum strain, which are expressed as $(\varepsilon_{max},\;\sigma_{max}),\;$$(\varepsilon_{h},\;\sigma_{hl}),\;$$(\varepsilon_{nh},\;\sigma_{nhl}),\;$$(\varepsilon_{min},\;\sigma_{min}),\;$$\left(\varepsilon_{nh},\;\sigma_{nhu}\right)$, and $(\varepsilon_{h},\;\sigma_{hu}),$ respectively. Moreover, by considering the normalization of input data for an ML model, the stress values in six points are further rewritten as dimensionless $\sigma_{max}/E,$ $\sigma_{hl}/E,\;$ $\sigma_{nhl}/E,$ $\sigma_{min}/E,$ $\sigma_{nhu}/E$, and $\sigma_{hu}/E,$ where *E* stands for the Young’s modulus of Q235B. It can be seen from the six points in Figure 7 that the strain value of $\varepsilon_{nh}$ or $\varepsilon_{h}$ corresponds to two points. If the stress and the corresponding strain in Cartesian coordinates are directly used as the input data of ML models, the points cannot be distinguished for characterizing hysteresis loops. In addition, the input data of ML models are always normalized to improve their performance, meaning that the negative strain values should be addressed. Therefore, we change the Cartesian coordinates of the six points to polar coordinates as shown in Figure 8. That is, we have the following transformations:

(1) $$\begin{array}{cc} \; & P_{1}:\;\left(\varepsilon_{max},\;\frac{\sigma_{max}}{E}\right)\to \left(\sqrt{\varepsilon_{max}^{2}+\frac{\sigma_{max}^{2}}{E^{2}}},\;\arctan\frac{\sigma_{max}}{\varepsilon_{max}E}\right), \end{array}$$

(2) $$\begin{array}{cc} \; & P_{2}:\;\left(\varepsilon_{h},\;\frac{\sigma_{hl}}{E}\right)\to \left(\sqrt{\varepsilon_{h}^{2}+\frac{\sigma_{hl}^{2}}{E^{2}}},\;2\pi +\arctan\frac{\sigma_{h}}{\varepsilon_{h}E}\right), \end{array}$$

(3) $$\begin{array}{cc} \; & P_{3}:\;\left(\varepsilon_{nh},\;\frac{\sigma_{nhl}}{E}\right)\to \left(\sqrt{\varepsilon_{nh}^{2}+\frac{\sigma_{nhl}^{2}}{E^{2}}},\;\pi +\arctan\frac{\sigma_{nhl}}{\varepsilon_{nh}E}\right), \end{array}$$

(4) $$\begin{array}{cc} \; & P_{4}:\;\left(\varepsilon_{min},\;\frac{\sigma_{min}}{E}\right)\to \left(\sqrt{\varepsilon_{min}^{2}+\frac{\sigma_{min}^{2}}{E^{2}}},\;\pi +\arctan\frac{\sigma_{min}}{\varepsilon_{min}E}\right), \end{array}$$

(5) $$\begin{array}{cc} \; & P_{5}:\;\left(\varepsilon_{nh},\;\frac{\sigma_{nhu}}{E}\right)\to \left(\sqrt{\varepsilon_{nh}^{2}+\frac{\sigma_{nhu}^{2}}{E^{2}}},\;\pi +\arctan\frac{\sigma_{nhu}}{\varepsilon_{nh}E}\right), \end{array}$$

(6) $$\begin{array}{cc} \; & P_{6}:\;\left(\varepsilon_{h},\;\frac{\sigma_{hu}}{E}\right)\to \left(\sqrt{\varepsilon_{h}^{2}+\frac{\sigma_{hu}^{2}}{E^{2}}},\;\arctan\frac{\sigma_{h}}{\varepsilon_{h}E}\right). \end{array}$$

When the early *k* hysteresis loops under a strain amplitude are considered, we can obtain 6k points. In addition, since four strain amplitudes are used for fatigue tests of Q235B here, there are a total of 4×6k points.

Second, the existing uncertainty in fatigue tests is quantified as an interval. On the one hand, we consider that the axial sensitivity of the extensometer of length 25 mm for the used MTS809 is ±0.002 mm. Therefore, each strain amplitude is extended to an interval as given in Table 4 [17]. On the other hand, it is considered that three specimens are tested under each strain amplitude. The interval logarithmic fatigue lives are determined and given in Table 4 by using the maximum–minimum method. Similarly, the maximum–minimum method is used to expand the 6k points in hysteresis loops under each strain amplitude to 2×6k intervals, which are written as ${\bar{D}}_{1}-{\bar{D}}_{4}$ in Table 4 by considering the four strain amplitudes, respectively.

Third, we randomly generate a great deal of data in each interval to construct the dataset. For example, by considering the strain amplitude 0.004 and the normal distribution, 50 points are randomly generated in a strain amplitude interval [0.00392, 0.00408], each polar coordinate interval in ${\bar{D}}_{1}$, and fatigue life interval [3.9129, 3.9946], respectively. Then, 50 vectors are constructed and represented in the following form:

(7) $${\vec{C}}_{1}=\left(s_{a}^{1};\;x_{1}^{1},\;g_{1}^{1},\ldots ,x_{6k}^{1},\;g_{6k}^{1};\;N_{f}^{1}\right).$$

Here, the symbol $s_{a}^{1}$ is the randomly generated strain amplitude; $x_{i}^{1}$ and $g_{i}^{1}$ (i∈{1, 2,…,6k}) stand for the values of the polar axis and the polar angle; $N_{f}^{1}$ denotes the fatigue life. Similarly, when we consider the strain amplitude 0.005, 0.006, and 0.008, 50 vectors can also be randomly generated, and represented by ${\vec{C}}_{2},\;$${\vec{C}}_{3}$ and ${\vec{C}}_{4},$ respectively. Thus, the dataset for ML models is constructed and written in the following form:

(8) $$X=\left\{{\vec{C}}_{1},\;{\vec{C}}_{2},\;{\vec{C}}_{3},\;{\vec{C}}_{4}\right\}.$$

When 50 points are randomly generated in each interval, there are 200 samples in X due to four strain amplitudes.

## 3. Machine Learning Models

Once the dataset is established by selecting feature points in stress–strain hysteresis loops under uncertainty, three ML models are developed for point predictions and interval predictions of fatigue life.

### 3.1. Back-Propagation Neural Network

**Figure 9:**
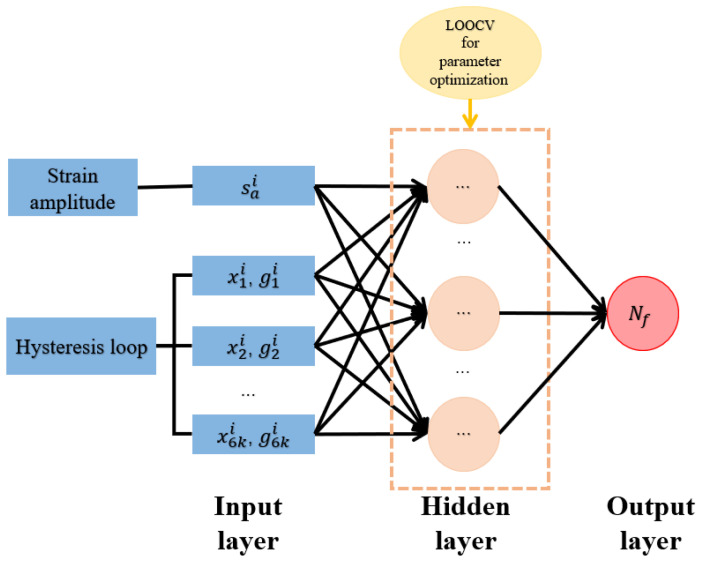
BP neural network model driven by the dataset X.

The BP neural network is the most commonly used model and has good nonlinear mapping ability. There are always the input layer, the hidden layers, and the output layer in a BP neural network, as shown in Figure 9. Each layer contains a certain number of neurons, which are not connected to each other. The neurons in adjacent layers are fully connected with weights and activated by using an activation function f(x) such as the Sigmoid function:

(9) $$f\left(x\right)=\frac{1}{1+e^{-cx}},$$

where the term *c* is a positive constant. The calculation of the BP neural network mainly consists of two parts: the forward-propagation of the input information and the back-propagation of the calculation error. The output of a neuron is expressed as:

(10) $$y_{j}=f\left(\sum_{i=1}^{m}w_{ij}x_{i}+b_{j}\right),$$

where $w_{ij}$ is the weight of the *j*-th neuron connected to the previous layer; $x_{i}$ is the value of the *i*-th neuron in the previous layer, and $b_{j}$ is the threshold.

To quantify the prediction accuracy, the coefficient of determination $R^{2}$ and mean squared error (MSE) are usually used. The formulae of $R^{2}$ and MSE are given below:

(11) $$\begin{array}{cc} R^{2}\left(y,\;y^{pre}\right) & =1-\frac{\sum_{i=1}^{n}{\left(y_{i}-y_{i}^{pre}\right)}^{2}}{\sum_{i=1}^{n}{\left(y_{i}-y_{mean}\right)}^{2}}, \end{array}$$

(12) $$\begin{array}{cc} MSE(y,\;y^{pre}) & =\frac{1}{n}\sum_{i=1}^{n}{\left(y_{i}-y_{i}^{pre}\right)}^{2}. \end{array}$$

Here, the term $y_{i}$ stands for the *i*-th experimental value; $y_{i}^{pre}$ is the *i*-th predicted value, and $y_{mean}$ is the mean value of experimental values. It can be seen that the closer the value of $R^{2}$ is to 1, or the smaller the value of MSE, the more accurate the predictions are. In addition, the coverage ratio of prediction interval (PICR) is utilized to describe the ratio for the predicted values that belong to a specific interval:

(13) $$PICR=\frac{1}{K}\sum_{i=1}^{K}\varphi_{i},$$

where *K* is the number of specimens and $\varphi_{i}=1$ means that the predicted value falls into the interval; otherwise, $\varphi_{i}=0.$

Prior to the application of the BP neural network model, we should determine the numbers of hidden layers and neurons. In the present study, by considering the small dataset X, leave-one-out cross validation (LOOCV) is used to give a stable and effective BP neural network. That is, when we consider 200 samples in X, the LOOCV has the following steps:

**Step** **1:** One sample is selected to construct the test set and the others are used to construct the training set.
**Step** **2:** The BP neural network model is carried out to give the MSE.
**Step** **3:** When each sample has been used as the test set, the average value of 200 MSEs is considered as the performance evaluation of the model.
**Step** **4:** The above steps are used for different BP neural network models and the best one is selected according to their performance evaluations.

Based on the above procedure, it is found that when the number of hidden layers is three, the numbers of neurons are determined as 18, 13, and 21 from the first hidden layer to the third hidden layer. The obtained neural network model will be used to predict the low-cycle-fatigue life of Q235B in the following section.

### 3.2. Support Vector Regression

**Figure 10:**
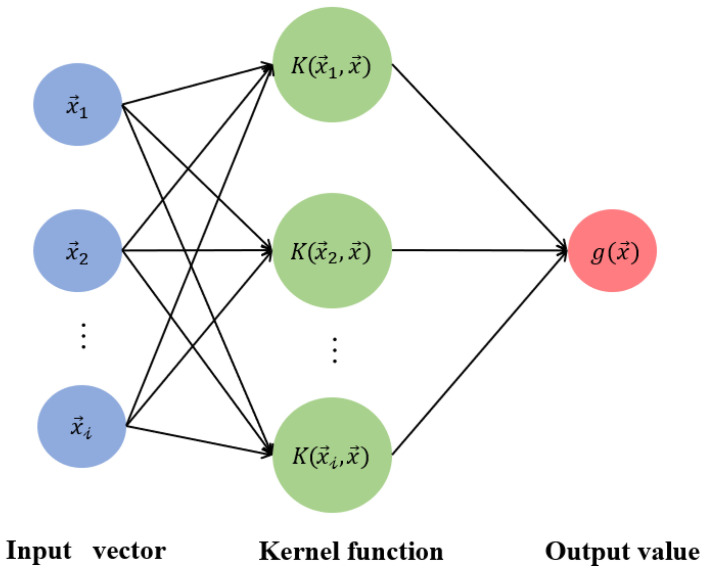
Flowchart of SVR.

SVR has obvious advantages in nonlinear mapping and high-dimensional pattern recognition. The key idea of SVR is to find a hyperplane that minimizes the distances from all sample points to this plane. Since the output of SVR for predicting fatigue life is a continuous value, an ε-insensitive loss function is introduced to achieve regression analysis. As shown in Figure 10, the ε-SVR determines a function $g\left(\vec{x}\right)={\vec{\omega }}^{T}\cdot \vec{x}+b$ such that each sample $\left({\vec{x}}_{i},\;y_{i}\right)$ in the training set is as close as possible to $g(\vec{x}).$

Letting the maximum allowable error be ε, the ε-insensitive loss function $l_{\varepsilon }$ is given as follows:

(14) $$l_{\varepsilon }=\left\{\begin{array}{cc} 0, & |g\left({\vec{x}}_{i}\right)-y_{i}|\;<\;\varepsilon ,\\ |g\left({\vec{x}}_{i}\right)-y_{i}|-\varepsilon , & \mathrm{otherwise}. \end{array}\right.$$

**Figure 11:**
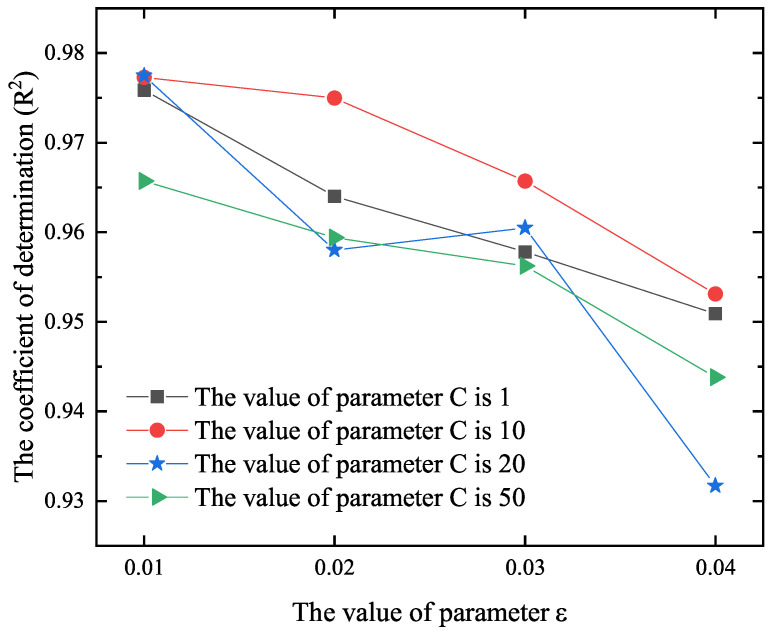
Variations of $R^{2}$ versus ε by selecting C=1, 10, 20, and 50, respectively.

Therefore, an optimization model is formed below:

(15) $$\min_{\vec{\omega },b}\frac{1}{2}{∥\vec{\omega }∥}^{2}+C\sum_{i=1}^{N}l_{\varepsilon },$$

where *N* is the number of samples in the training set and *C* is the regularization parameter or penalty coefficient. Once the weight vector $\vec{\omega }$ and *b* are determined, the regression function $g(\vec{x})$ is used for prediction. When the training set is not linearly separable, a nonlinear kernel function is always introduced to transform the dataset to a high-dimensional space to make it linearly separable. In the present study, the radial basis function is selected as the kernel. In order to determine the parameters ε and C, we consider the variations of $R^{2}$ in Figure 11. It is found that when ε=0.01 and C=10, the prediction accuracy is the highest.

### 3.3. Random Forest

**Figure 12:**
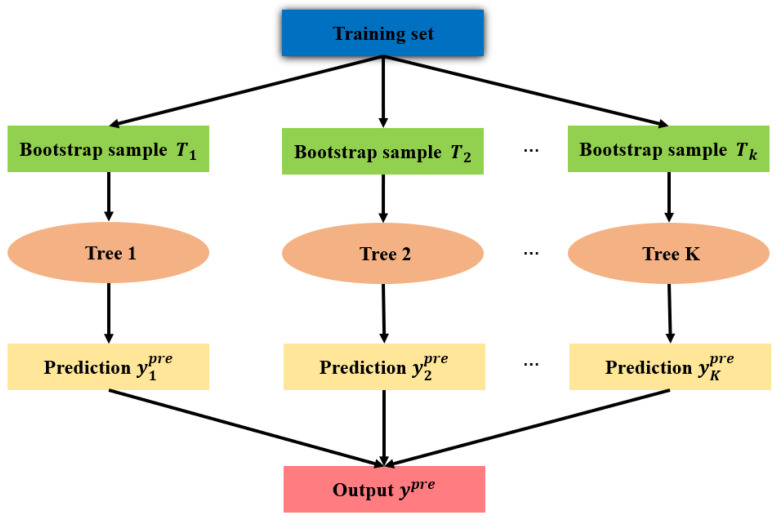
Network structure of RF.

RF is an ML algorithm based on statistical theory and ensemble learning strategy. The network structure of an RF model is shown in Figure 12. The Bootstrap sampling method is used to extract multiple sample sets from the training set to construct multiple decision trees. Each decision tree is applied to give an independent prediction. The final prediction is determined by voting or averaging. In the present study, assume that the training set of each decision tree is randomly selected as $T_{k}=\left\{\left({\vec{x}}_{1}^{k},\;y_{1}^{k}\right),\ldots ,\left({\vec{x}}_{l}^{k},\;y_{l}^{k}\right)\right\}$, where ${\vec{x}}_{i}^{k}=\left(x_{i}^{1},\;x_{i}^{2},\ldots ,x_{i}^{n}\right)$ is an input vector with *n* elements, and $y_{i}^{k}$ is the output value. If RF contains K≥1 decision trees and the predicted values are $y_{i}^{pre}$ (i=1,2,…,K), the final predicted value $y^{pre}$ is calculated as:

(16) $$y^{pre}=\frac{1}{K}\sum_{i=1}^{K}y_{i}^{pre}.$$

**Figure 13:**
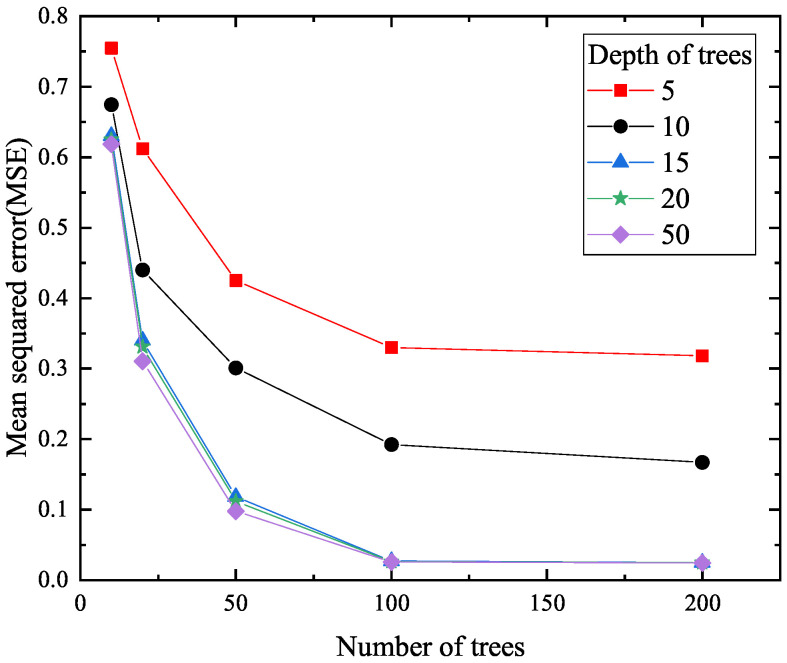
The effects of the number of trees $n_{t}$ and the depth of trees $n_{d}$ on MSE.

When RF is used, the number of trees $n_{t}$ and the depth of trees $n_{d}$ should be selected carefully. Figure 13 is drawn to show the effects of $n_{t}$ and $n_{d}$ on MSE. It is found from Figure 13 that when the depth of trees is fixed, the values of MSE decrease with the increasing number of trees under $n_{t}\leq 100.$ When $n_{t}\geq 100,$ the value of MSE is changed slightly for a fixed depth of trees. Moreover, when the number of trees is fixed as $n_{t}\leq 100,$ the values of MSE drop down. Observation reveals that the values of $n_{t}=100$ and $n_{d}=15$ are optimal by considering the computational cost. For convenience, the hyperparameters used in the three ML models are summarized in Table 5.

## 4. Results and Discussion

Based on the developed ML models, we predict the low-cycle-fatigue life of Q235B by using the constructed dataset X. As shown in Table 4, the training dataset is formed by considering the strain amplitudes 0.004, 0.005 and 0.006; and the test dataset is based on the strain amplitude 0.008. In order to illustrate the effects of the input data on the predictions, four cases in Table 6 are considered. In case 1, the input data include the strain amplitude and the selected six points at each hysteresis loop of the earliest 10 cycles of fatigue process. This implies that the dimension of input data in case 1 is 61. Similarly, we can see that the dimensions of input data in cases 2, 3, and 4 are 31, 60, and 30, respectively. Moreover, in each interval of X, 50 data points are randomly generated to strain the developed ML models and carry out the point/interval predictions of the low-cycle-fatigue life of Q235B.

### 4.1. Point and Interval Predictions Based on BP Neural Network Model

**Figure 14:**
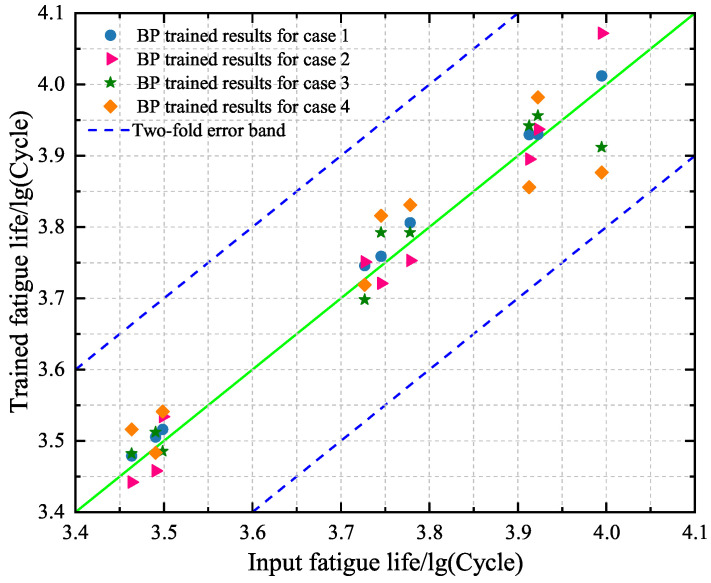
Comparison between the predicted values and the experimental results of the training set based on the trained BP neural network models for cases 1–4, respectively.

In the following, the BP neural network model is used, where there are three hidden layers with 18, 13, and 21 neurons. The performance of the strained BP model on the training set is shown in Figure 14 by comparing the predicted values and the experimental data of fatigue life. It is seen from Figure 14 that all the points for four cases are located in the two-fold error band and approximate to the diagonal. This means that there is good accuracy for the training set when using the BP neural network model. A comparison between the four cases reveals that a point in case 4 is further away from the diagonal than the other points. The phenomenon shows that the performance of the trained BP model for case 4 is slightly worse than the other cases.

**Figure 15:**
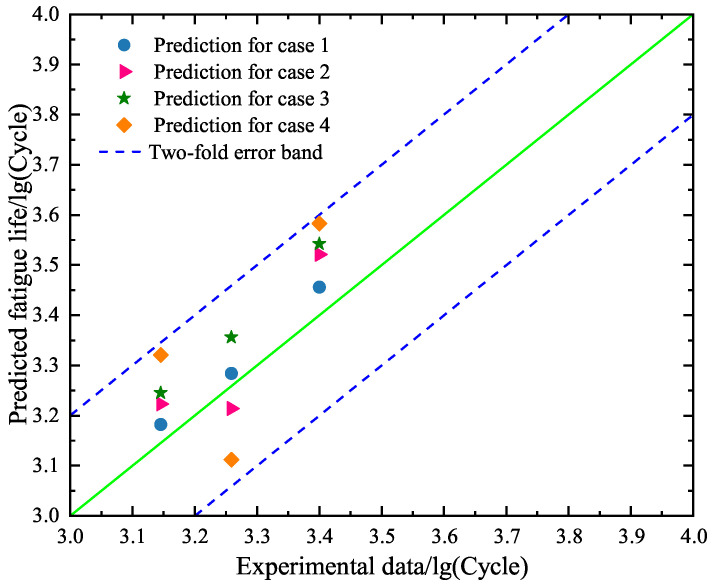
Comparison between the predicted values and the experimental results of the test set based on the trained BP neural network models for cases 1–4, respectively.

Furthermore, we use the trained BP neural network models for the four cases to predict the low-cycle-fatigue lives of specimens A10–A12 under the strain amplitude of 0.008. Figure 15 is drawn to show the comparison between the predicted values and the experimental data by using the trained BP neural network models for cases 1–4, respectively. We can find from Figure 15 that the predicted values all belong to the two-fold error band. Observation reveals that the hysteresis loop-based BP neural network model is effective with regards to giving an acceptable prediction of fatigue life. We further compare cases 1–4 and find that the obtained results of case 1 are the most accurate. A comparison between case 1 and case 2 shows that the use of the early 10 hysteresis loops is better than the early 5 hysteresis loops. A comparison between case 1 and case 3 reveals that the strain amplitude plays an important role in the prediction of fatigue life. When the influence of the strain amplitude on the prediction of fatigue life is neglected, the results of cases 3 and 4 are still acceptable for the developed models.

**Figure 16:**
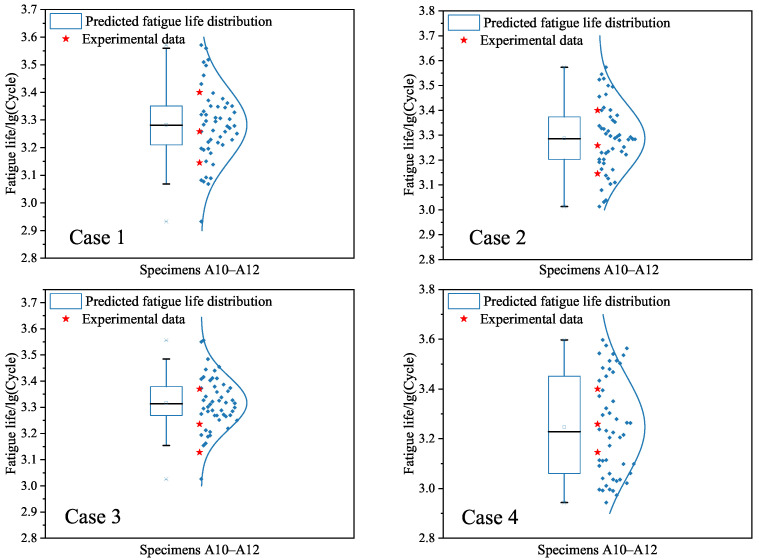
Interval predictions of logarithm fatigue life for specimens A10–A12 based on the BP neural network models for cases 1–4, respectively. The blue points are the predicted values based on the randomly generated input data.

At the end, we focus on the dispersion of fatigue life since the same specimen configuration yields different fatigue test lives under the same strain amplitude. Based on the uncertainty quantification as shown in Table 4, the randomly generated data are used to predict the interval-valued logarithm fatigue lives of specimens A10–A12 as given in Figure 16. It is found from Figure 16 that the mean values of the predicted logarithm fatigue lives are all located in the interval [3.1452, 3.4]. Comparisons between cases 1–4 indicate that the mean value of case 1 is less than those of cases 2–4. The variability for the four cases is significant, since the values of many points are less than 3.1452 or more than 3.4. The underlying reason is that the input data are completely randomly generated. In order to more accurately characterize the dispersion of predictions, some physical knowledge should be used to capture the relationships between the input data and the fatigue life.

### 4.2. Point and Interval Predictions Based on SVR Model

**Figure 17:**
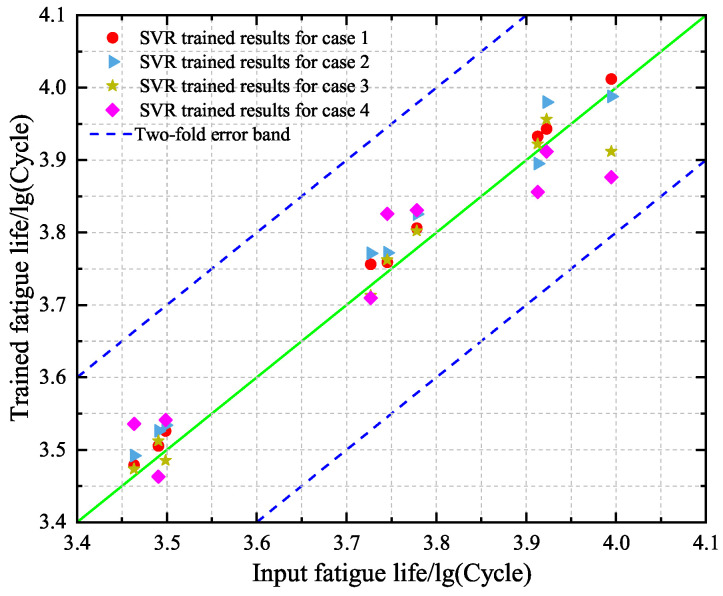
Comparison between the predicted values and the experimental results of the training set based on the constructed SVR models for cases 1–4, respectively.

Now, the SVR model with ε=0.01 and C=10 is adopted to give point and interval predictions of the low-cycle-fatigue life of Q235B. First, the training set including specimens A1–A9 is used to construct the SVR models under cases 1–4, respectively, and the performance is given in Figure 17. A comparison between the predicted values and the experimental data for cases 1–4 shows that the fatigue lives of A1–A9 have been evaluated correctly by using the SVR models. This means that the developed SVR models have been constructed reasonably and can be used for predictions.

**Figure 18:**
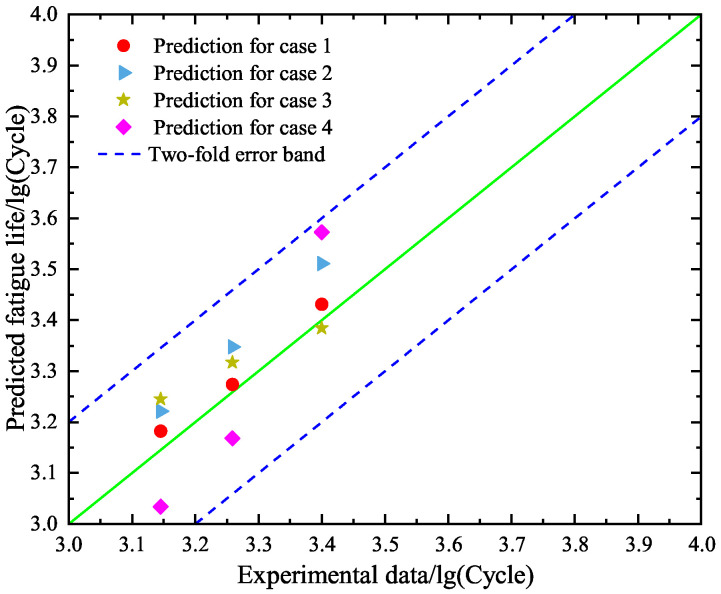
Comparison between the predicted values and the experimental results of the test set based on the constructed SVR models for cases 1–4, respectively.

Second, the constructed SVR models under cases 1–4 are used to predict the fatigue lives of specimens A10–A12 in the test set. The obtained results are given in Figure 18, where the predicted values and the experimental data are compared. One can see that the predicted results under case 1 perform the best and those under case 4 are the worst. This phenomenon is in agreement with the finding for the BP neural network model. By comparing Figure 15 and Figure 18, it is found that the prediction accuracy of the SVR model is higher than the BP neural network model.

**Figure 19:**
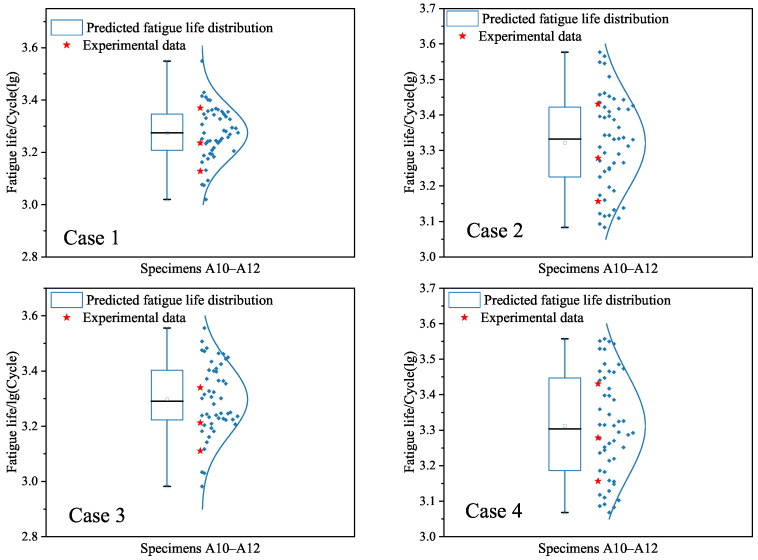
Interval predictions of logarithm fatigue life for specimens A10–A12 based on the SVR models for cases 1–4, respectively. The blue points are the predicted values based on the randomly generated input data.

Third, we apply the constructed SVR models under cases 1–4 to give interval predictions of the low-cycle-fatigue lives of specimens A10–A12. Figure 19 is depicted to show the probability distributions of the predicted values under the four cases. It can be seen from Figure 19 that the dispersion of fatigue life is still high due to the randomness of generated data. This observation is similar to the findings in Figure 16 based on the BP neural network model. The difference is the mean values of the predicted fatigue life in Figure 19, which are less than those in Figure 16 under cases 1–4, respectively.

### 4.3. Point and Interval Predictions Based on RF Model

**Figure 20:**
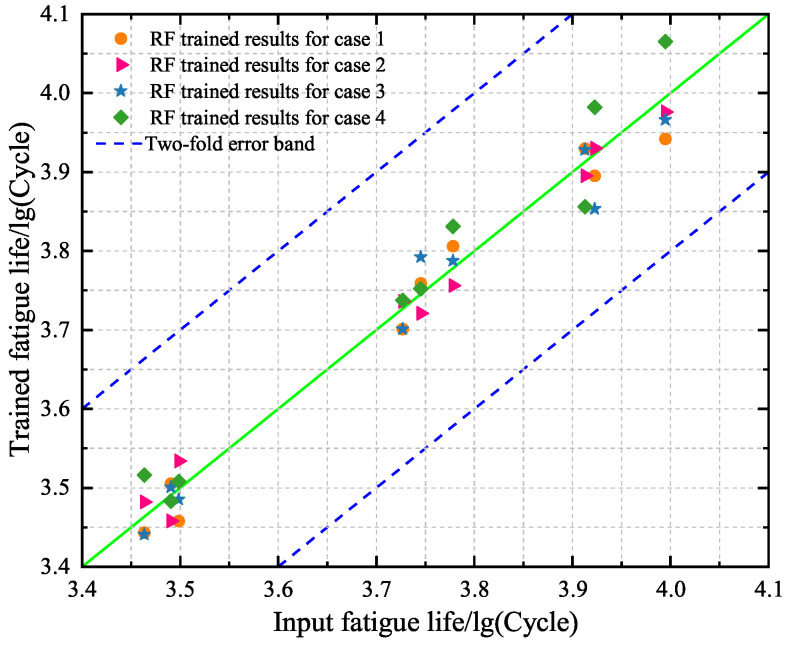
Comparison between the predicted values and the experimental data of the training set based on the selected RF models for cases 1–4, respectively.

In what follows, the RF model with $n_{t}=100$ and $n_{d}=15$ is selected to predict the fatigue lives of specimens A10–A12. First, the performance of the trained RF model on the training set is investigated under cases 1–4 and is shown in Figure 20. The obtained results show that the points are distributed around the diagonal. This means a good accuracy of the computed fatigue lives of specimens A1–A9 for cases 1–4, respectively. As compared to Figure 14 and Figure 17, it is revealed that the ML models can all be trained effectively.

**Figure 21:**
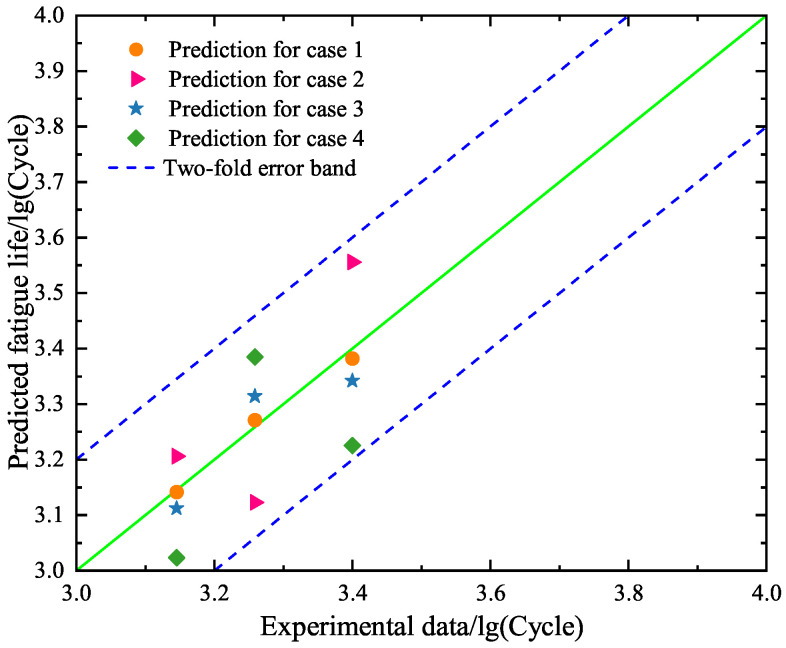
Comparison between the predicted values and the experimental data of the test set based on the selected RF models for cases 1–4, respectively.

Second, we predict the fatigue lives of specimens A10–A12 based on the RF models under cases 1–4. As shown in Figure 21, the predicted fatigue lives are all located at the two-fold error band, meaning that the RF models are effective. A comparison between cases 1–4 reveals that the predicted values under case 1 are the most accurate. The observation is in accordance with the findings based on the BP neural network model and the SVR model. The novel finding in Figure 21 is that the predicted results under cases 1 and 3 are better than those under cases 2 and 4. This means that the RF model is more influenced by the dimensionality of input data. In addition, we compare Figure 21 with Figure 15 and Figure 18 to find that the accuracy of the RF model is higher than the BP neural network model and the SVR model for case 1.

**Figure 22:**
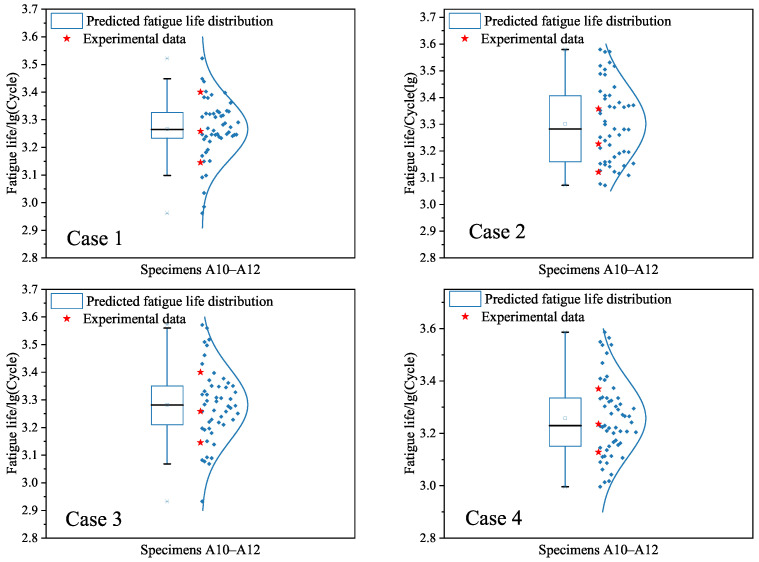
Interval predictions of logarithm fatigue life for specimens A10–A12 based on the RF models for cases 1–4, respectively. The blue points are the predicted values based on the randomly generated input data.

Third, we provide the interval predictions of fatigue lives of specimens A10–A12 based on the RF models in Figure 22. One can see from Figure 22 that the dispersion of fatigue life is captured by considering the uncertainty quantification and using the randomly generated data. The dispersion degree of the predicted fatigue lives is approximate to those in Figure 16 under the BP neural network model and Figure 19 under the SVR model. To further show the difference between the developed ML models, the mean values and variances of probability distributions in interval predictions are given in Table 7. This finding reveals that the randomness of generated data is the main factor affecting the variability of predictions. The appropriate physical knowledge should be used to improve the characterization of dispersion of fatigue life predictions.

In summary, the developed ML models can be used to effectively give point predictions of fatigue life of Q235 by constructing the hysteresis loop-based dataset. By considering the uncertainty quantification, the interval predictions are provided to characterize the dispersion in fatigue life prediction. As compared to the models in [17], the novelty comes from the method of constructing the dataset. Different from the approach in [31], here we only use the 10 hysteresis loops at the earliest stages of the fatigue process to give a good prediction. As shown in [34], it is convenient to compare the performances of three models for point predictions of fatigue life in Table 8. The changes in performance of ML models are statistically significant by considering the influences of the data extracted from stress–strain hysteresis loops. The basic reason is that the dispersion of fatigue life is attributed to the difference in stress–strain hysteresis loops for the same specimen. The statistically significant can be used for future studies, when the implicit physical mechanism in stress–strain hysteresis loops is characterized by the extracted data.

## 5. Conclusions

Understanding fatigue behavior of metallic materials plays an important role in engineering. The fatigue strength is an important parameter to characterize the fatigue behavior. Under different fatigue loadings, the fatigue life of materials should be evaluated for the safety of engineering structures. In this paper, we have considered the dependence of metal fatigue life on the stress–strain hysteresis loops. Three machine learning models—the back-propagation neural network model, the support vector regression model, and the random forest model—were developed to predict the low life fatigue of Q235B. Some interesting conclusions are worth mentioning below:

(a) The constructed dataset based on the early 10 hysteresis loops is effective at characterizing the fatigue features under different strain amplitudes.
(b) The challenge of small fatigue test datasets has been addressed to a certain degree according to uncertainty quantification.
(c) Random forest is more accurate than the other two models for point predictions of fatigue life by considering 10 hysteresis loops and strain loadings.

Our work has provided a novel perspective for predictions of metal fatigue life by considering the dependence on the stress–strain hysteresis loops at the earliest stages of the fatigue process. However, there are still some opportunities for further research concerning the following: (1) How to physically interpret the randomly generated data to enhance the reliability of the trained machine learning models. (2) How to characterize the dependence of fatigue life on the feature points used in machine learning models. (3) How to develop physics-informed machine learning models to capture the dispersion in fatigue life prediction. (4) How to reveal the fatigue mechanism according to hysteresis loop-based machine learning models for predicting fatigue life.

## Figures and Tables

**Table 1:** Mechanical properties of Q235B steel.

Yield Strength/Mpa	Ultimate Strength/Mpa	Young’s Modulus/Mpa	Poisson’s Ratio	Elongation
260	550	193900	0.277	20%

**Table 2:** Main chemical compositions of Q235B steel (%).

*C*	Si	Mn	*P*	*S*	As	Cep
0.14	0.032	0.4	0.03	0.019	0.031	0.42

**Table 3:** Experimental data of Q235B.

Axial Strain Amplitude							
0.004		0.005		0.006		0.008	
Specimen	$\mathrm{lg}N_{f}$	Specimen	$\mathrm{lg}N_{f}$	Specimen	$\mathrm{lg}N_{f}$	Specimen	$\mathrm{lg}N_{f}$
A1	3.9227	A4	3.7782	A7	3.4905	A10	3.2586
A2	3.9129	A5	3.7455	A8	3.464	A11	3.1452
A3	3.9946	A6	3.7269	A9	3.4984	A12	3.4

**Table 4:** Interval-valued data of Q235B smooth specimens.

Class	Specimen	Strain Amplitude Interval	Hysteresis Loop	Fatigue Life (lg)
Training set	A1, A2, A3	[0.00392, 0.00408]	${\bar{D}}_{1}$	[3.9129, 3.9946]
	A4, A5, A6	[0.00492, 0.00508]	${\bar{D}}_{2}$	[3.7269, 3.7455]
	A7, A8, A9	[0.00592, 0.00608]	${\bar{D}}_{3}$	[3.464, 3.4984]
Test set	A10, A11, A12	[0.00792, 0.00808]	${\bar{D}}_{4}$	[3.1452, 3.4]

**Table 5:** Optimized hyperparameters in ML models.

ML Model	Hyperparameter	Value
BP	Number of hidden layers	3
	Numbers of neurons in hidden layers	18, 13, 20
	Learning rate	0.005
	Activation function	Sigmoid
SVR	Kernel	Radial basis function
	Maximum allowable error	ε=0.01
	Regularization parameter	C=10
RF	Number of trees	$n_{t}=100$
	Depth of trees	$n_{d}=15$

**Table 6:** Four cases of input data.

Case	Strain Amplitude?	Number of Early Hysteresis Loops	Dimension of Input Data
Case 1	Yes	10	61
Case 2	Yes	5	31
Case 3	No	10	60
Case 4	No	5	30

**Table 7:** Mean values and variances of probability distributions in interval predictions.

Case	BP Model		SVR Model		RF Model	
	Mean	Variance	Mean	Variance	Mean	Variance
Case 1	3.28	0.032	3.27	0.034	3.23	0.030
Case 2	3.27	0.036	3.29	0.038	3.22	0.038
Case 3	3.30	0.042	3.25	0.041	3.31	0.036
Case 4	3.23	0.064	3.22	0.068	3.30	0.062

**Table 8:** Performances of three models for fatigue life point predictions of Q235B.

Case	BP Model	SVR Model	RF Model
	MSE	MSE	MSE
Case 1	0.0011	0.0041	0.0002
Case 2	0.0058	0.0069	0.0149
Case 3	0.0108	0.0119	0.0025
Case 4	0.0260	0.0155	0.0202

## Data Availability

The original contributions presented in this study are included in the article. Further inquiries can be directed to the corresponding author.

## Abbreviations

The following abbreviations are used in this manuscript:

ML	Machine learning
BP model	Back-propagation neural network model
MSE	Mean squared error
PICP	The coverage ratio of prediction interval
RF	Random forest
SVM	Support vector machine
SVR	Support vector regression
